# The individuality of stem cells

**DOI:** 10.1186/1741-7007-9-40

**Published:** 2011-06-07

**Authors:** Arthur D Lander

**Affiliations:** 1 2638 Biological Sciences III, University of California Irvine, Irvine, CA 92697-2300, USA

## Comment

When a group of immigrants moves into a community in large numbers, so much attention is usually focused on how they are different - in language, customs, appearance, and so on - from everyone else, that little notice is taken of how different they may be from each other. It is only after some time that new immigrant groups tend to be seen as diverse sets of people defined by their individuality, and not merely by their shared group characteristics.

**Figure 1:**
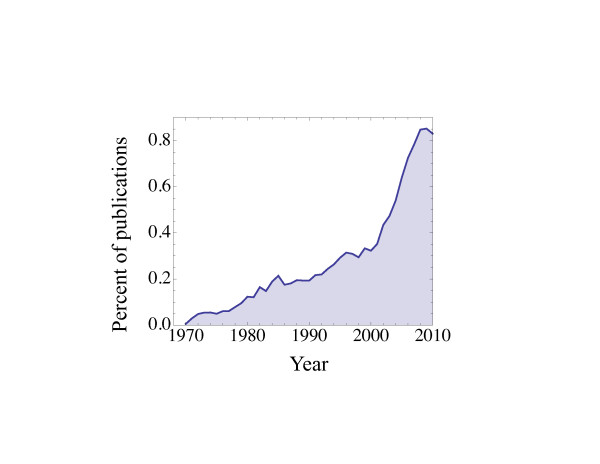
**Publications indexed on PubMed by MeSH major topic 'stem cell', from 1970 to 2010, as a percentage of total indexed publications**. Between 1995 and 2008, the rate of publication on stem cells increased threefold faster than the overall publication rate (which itself nearly doubled over the same period).

Similar things may be said about stem cells. Although not a new subject in biology, in the last decade and a half, stem cells seem truly to have exploded onto the scene of biological research (Figure 1). Not surprisingly, attitudes about stem cells have focused largely on the ways in which they are different from other cells. Thus, basic research on stem cells has been dominated by a search for explanations of properties thought to be common to stem cells, such as self-renewal, immortality, pluripotency and asymmetry of division. Yet in recent years, there has been growing awareness that such properties are not unique to stem cells, nor do all types of stem cells necessarily possess them, nor do those that possess them manifest them at all times. Such recognition that there is diversity and plasticity among types of stem cells has freed us to start paying closer attention to the diversity of behaviors displayed by individual stem cells, even within supposedly homogeneous groups.

## Do stem cells play dice?

Nowhere is such individuality more evident than in clonal-analysis studies, which involve the tracking of stem cells and their offspring over time. Clonal analysis has a long history in the stem-cell field, going back to pioneering work on hematopoietic stem cells in the early 1960s [1]. Such work has always suggested that stem cells behave stochastically - essentially rolling dice at each cell division to determine whether to make two progeny that are both stem cells, two progeny that are non-stem cells, or one of each [2]. Yet for years, most biologists have espoused a deterministic view, in which stem cells all behave in predetermined ways, usually dividing asymmetrically (at least under normal circumstances), to produce one stem cell and one 'transit-amplifying cell', which then replicates itself a fixed number of times before finally differentiating [3,4].

The widespread adoption by biologists of the deterministic, stem/transit-amplifying model should be seen less as an unwillingness to accept the possibility of stochastic stem-cell behavior than as an expression of hope that the degree of individuality that stem cells display is sufficiently small as to be negligible. Alas, that hope now appears to have been thoroughly dashed by a series of recent studies involving some of the most widely studied tissue stem-cell systems [5-7]. In one case - the mouse small intestine - direct observations indicate that the proportion of times that stem cells divide asymmetrically is astonishingly small, on the order of 20%; the rest of the time they choose equally between making either two stem cells or differentiating [7]. In other cases, such as mouse interfollicular epidermis, asymmetric divisions are more frequent, but still far from exclusive [5].

Rather than having a negligible impact, such behavior should produce highly characteristic and meaningful patterns of clonal dynamics. This is a reflection of the fact that every stem-cell division that produces two differentiated cells (symmetric differentiation) will extinguish a stem-cell clone, whereas every stem-cell division that produces two stem cells (symmetric renewal) will make a clone significantly less likely to be extinguished in the near future. Accordingly, if one were to track the behaviors of a population of stem cells in a tissue, one would observe that many undergo a small number of divisions before being absorbed into the pool of differentiated cells, whereas a subset seems to undergo division for a very long time without all differentiating. Remarkably, it was observations of precisely this sort in the epidermis that first led to the formulation of the stem/transit-amplifying model [3,4]. In other words, as has now been clearly pointed out [5], what was originally thought to be evidence for the existence of two distinct cell types (stem and transit-amplifying) is just as easily interpreted as evidence for a single cell type behaving stochastically. Not only must we accept the possibility that transit-amplifying cells do not exist, we have to face the fact that our reasons for believing in them in the first place may never have been very good.

Interestingly, the basic statistical arguments that make this point had been published more than a decade before the formulation of the stem/transit-amplifying model, in a series of theoretical papers motivated by the behaviors of hematopoietic clones (see, for example, [2,8]). Why this work had little impact on the community of researchers working on stem cells in solid tissues is unknown, but may reflect a traditional view among experimental biologists that one should resort to mathematical and statistical arguments only when more intuitive kinds of reasoning fail. Fortunately, such attitudes appear to be changing, perhaps as a result of increased recognition of the importance of stochastic phenomena in biology in general [9]. In this issue of *BMC Biology*, for example, Dingli and Pacheco [10] discuss the implications of stochastic stem-cell dynamics on the accumulation of mutations in stem-cell populations. They illustrate how such dynamics explain several clinically relevant phenomena, including the observed high rate at which certain kinds of acquired hematological disorders spontaneously cure themselves.

## Implications of stochastic stem-cell behavior

Further exploration of the relationships between stochastic stem-cell dynamics, mutation and natural selection in other organ systems is clearly warranted. One obvious question is whether there is an optimal relationship between the degree of division asymmetry that a stem-cell population exhibits, the size of the population, and the rates at which mutations accumulate or are flushed out by clonal extinction. Other questions concern the impact of ever-decreasing clonal diversity on stem-cell aging. Still others concern the impact, on mutation accumulation and aging, of the arrangement of stochastically behaving stem cells into clonal hierarchies, with 'resting' and 'active' stem cells that divide at very different rates.

In addition to its implications for the way in which heritable genetic changes accumulate, the stochastic behavior of stem cells has implications for the way in which tissue growth, homeostasis and regeneration are controlled. The reasons for this are simple: if the stem cells in a given tissue all behave alike - always dividing asymmetrically, always producing the right differentiated cell types at the right times - then there is much less that needs to be controlled than if such stem cells roll dice to make their decisions. For example, in a constantly turning-over tissue, homeostasis requires that the number of symmetric renewal divisions exactly equals the number of symmetric differentiation divisions, or else the tissue will either grow without limit or shrink to extinction. How can such equal loading of dice be ensured in every cell? It has recently been argued that feedback regulation of renewal probabilities by secreted molecules (chalones) must play a role in such control [11-13]. Not just renewal probabilities, but the fate choices of stem-cell progeny also seem to be regulated by feedback control [14,15]. Whether stem-cell division symmetry is itself the object of control is an open question, but the fact that symmetry proportions vary widely, but consistently, among tissues suggests that it may be [5,7].

The need and opportunity for multiple levels of control of stochastic stem-cell behaviors suggests a novel interpretation of the traditional concept of the stem cell 'niche'. Usually viewed as hospitable locations in which stem cells must reside in order to display their intrinsic characters, niches may turn out to have less to do with the need to keep stem cells in a stem-like state than with the need to achieve control and coordination over the intrinsic individuality of stem cells. In effect, stem cell niches may represent nature's way of stating that harmony within populations is more efficiently achieved by acknowledging, cultivating and managing individuality than by suppressing it. There is an obvious lesson in this for human populations. Indeed, it is a lesson that encounters with immigrant groups can, under the right circumstances, help us learn.
